# A case report and literature review: Identification of a novel *AIRE* gene mutation associated with Autoimmune Polyendocrine Syndrome Type 1 in East Asians

**DOI:** 10.1097/MD.0000000000020000

**Published:** 2020-05-01

**Authors:** Zi Yan, Xiaokun Gang, Xiaona Xie, Ying Gao, Zhuo Li, Guixia Wang

**Affiliations:** Department of Endocrinology and Metabolism, The First Hospital of Jilin University, Changchun, China.

**Keywords:** autoimmune polyendocrine syndrome type 1 (APS-1), autoimmune polyendocrinopathy candidiasis-ectodermal dystrophy, autoimmune regulator gene (*AIRE*), East Asians, mutation

## Abstract

**Rationale:**

Autoimmune polyendocrine syndrome type 1 (APS-1), also referred as the autoimmune polyendocrinopathy candidiasis-ectodermal dystrophy (APECED), is a rare autosomal inherited disease predominantly among Caucasians from Northern Europe. This syndrome is very rare in East Asian population.

**Patients concerns:**

Here, we describe a case of a 15-year-old Chinese boy admitted due to a 1-month history of intermittent fatigue, nausea, vomiting, and diarrhea. His symptom became worse accompanied with chest tightness 4 days before admission. On physical examination, his temperature was 38.5°C, blood pressure was 75/38 mm Hg, and pulse was 98/min. He was a thin boy with mild hyperpigmentation and xanthochromia.

**Diagnosis:**

After abdominal computed technology and laboratory tests, his diagnosis was APS-1 accompanied with adrenal crisis. Further investigation on whole-exome sequencing revealed a novel homozygous mutation c.47C>G (p.T16R) in exon 1 in the *autoimmune regulator* (*AIRE*) gene.

**Interventions:**

This patient underwent replacement therapy of glucocorticoids, corticosteroid, and levothyroxine, as well as calcium and calcitriol supplementation.

**Outcomes:**

He continues to do well 4 years after his hospitalization. During his last follow-up, he had serum thyroid-stimulating hormone level of 3.07 μIU/mL, free triiodothyronine level of 1.92 pg/mL, and free thyroxine level of 13.95 pg/mL. His serum cortisol and ACTH (8 a.m.) levels were 28.53 μg/dL and 69.48 pg/mL, respectively.

**Lessons:**

APS-1 is very rare in East Asians and the variable clinical presentations of the disease make the initial diagnosis especially difficult. Autoimmune thyroiditis, type 1 diabetes mellitus, and hepatitis were the three most frequent minor components of APS-1 in East Asian patients with age of onset in late teens and 20s. Sequence analysis of *AIRE* gene is necessary to verify its diagnostic efficacy in association with clinical findings.

## 1 Introduction

Autoimmune polyendocrine syndrome type 1 (APS-1), also known as the autoimmune polyendocrinopathy-candidiasis-ectodermal dystrophy (OMIM 240300) is a rare monogenic inherited disorder caused by mutation of human autoimmune regulator (*AIRE*) gene located on chromosome 21q22.3.^[1]^ Diagnosis of this syndrome requires the presence of at least two of the classical triads: chronic mucocutaneous candidiasis (CMC), hypoparathyroidism and Addison's disease (AD), or only one major component if a sibling is affected by APS-1.^[2]^ Other autoimmune endocrinopathies such as autoimmune thyroid diseases, hypertrophic hypogonadism, and type 1 diabetes mellitus (T1D), as well as various non-endocrine features have been reported to be associated with APS-1 such as ectodermal dystrophy (alopecia, vitiligo, nail dystrophy, dental enamel dysplasia, and keratopathy), autoimmune or immuno-mediated gastrointestinal diseases (malabsorption, diarrhea, autoimmune gastritis, and pernicious anemia), chronic active hepatitis or autoimmune hepatitis, asplenia, and hemolytic anemia.

To date, 129 different mutations of *AIRE* gene have been identified in patients with APS-1 from various countries, predominantly in some genetically isolated populations such as Iranian Jews (1:9000),^[3]^ Sardinians (1:14,400),^[4]^ and Finns (1:25,000),^[5]^ while scarce in East Asians. A rough estimate incidence of the disease is 1:10,000,000 in Japan.^[6]^ In the present study, the authors describe a 15-year-old Chinese boy with clinically diagnosed APS-1 caused by a novel missense mutation in the *AIRE* gene, and reviewed literatures on APS-1 in the East Asian population.

## 2 Case presentation

A 15-year-old Chinese boy of consanguineous parents of first cousins was referred to the authors with a 1-month history of intermittent fatigue, nausea, vomiting, and diarrhea. His symptom became worse accompanied with chest tightness 4 days before admission. He had a history of frequent relapse of oral candidiasis at age 3. On physical examination, his temperature was 38.5°C, blood pressure was 75/38 mm Hg, and pulse was 98/min. He was a thin boy with mild hyperpigmentation and xanthochromia. Laboratory test results revealed hyponatremia (121.1 mEq/L), hypochloridemia (87.2 mEq/L), hyperkalemia (5.79 mEq/L), and hypoglycemia (51.48 mg/dl). His serum cortisol (8 a.m.) level was 0.64 μg/dL, 24-h urinary-free cortisol value was 22.5 μg, and serum adrenocorticotropic hormone (ACTH) level (8 a.m.) was 676.20 pg/mL. Initial diagnosis of AD accompanied by adrenal crisis was made. In addition, laboratory tests revealed hypoparathyroidism evidenced by hypocalcemia (1.93 mEq/L), hyperphosphatemia (2.63 mEq/L), and a low parathromone level (<1 pg/mL). An elevated thyroid-stimulating hormone level (12.82 μIU/mL) and a low free thyroxin level (1.72 pg/mL), together with positive antithyroglobulin (4000 IU/mL) and antithyroid microsomal antibodies (534.2 IU/mL) suggested the presence of autoimmune thyroiditis. Routine blood test was normal. Liver function test showed elevated serum aspartate aminotransferase (45.7 U/L), serum total bilirubin (9.68 mg/dL), and serum unconjungated bilirubin (9.34 mg/dL). Considering the autoimmune nature of the suspected disease, autoimmune hemolysis associated tests (serum hemolysis test, plasma free hemoglobin, and Coombs test) were performed, but the results were negative. Hemolysis was further ruled out by peripheral blood smear showing only poikilocytosis, but not Howell-Jolly bodies, thrombocytosis, anysocites, target cells, or burr cells. Tests on organ-specific autoantigens associated with APS-1 were performed. Antinuclear antibody, anti-neutrophil cytoplasmic antibodies were negative. Insulin-dependent diabetes mellitus was excluded by normal serum glucose, insulin, and C-peptide level, as well as absence of autoimmune antibodies associated with T1D (islet cell antibody, glutamic acid decarboxylase antibody, insluinoma-associated 2 molecule antibody and insulin autoantibody). Autoantibodies against autoimmune hepatitis (liver cytosol antibody type 1 antibody, soluble liver antigen/liver-pancreas antibody, gp210 antibody, and liver/kidney/microsomal antibody) and primary biliary cirrhosis (PML antibody, sp100 antibody, M2-3E antibody, and M2 antibody) were undetectable. Abdominal computed tomography (CT) demonstrated a morphologically small spleen and cholestasis. Normal adrenal glands and pituitary gland was identified via CT and Magnetic Resonance Imaging, respectively. Binocular crystals were clear.

**Figure 1:**
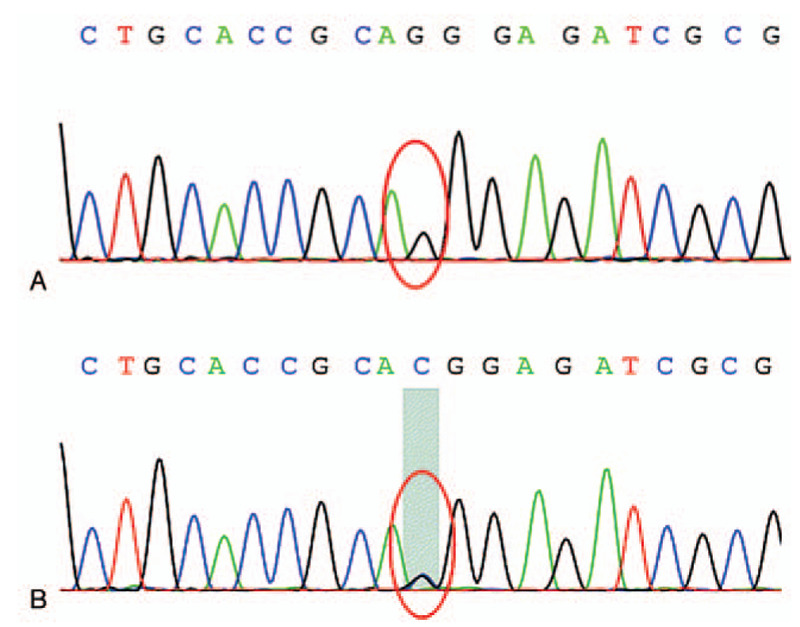
Chromatogram of the c.47C>G mutation. (A) Whole-exome sequencing revealed a homozygous C to G mutation (*red circle*) at position 47 in exon 1 of *AIRE* gene in the proband. This leads to a missense mutation by a substitution of threonine with arginine at amino acid position 16 (p.T16R). (B) The same heterozygous c.47>G (*red circle*) mutation was identified in his parents.

The concomitant diagnosis of AD and hypoparathyroidism with the history of oral candidiasis fulfilled the clinical diagnostic criteria for APS-1. The patient also suffered from autoimmune thyroiditis and spleen atrophy, which are the minor components of APS-1. Further investigation on whole-exome sequencing of the entire protein-coding region of *AIRE* gene revealed a novel homozygous mutation (c.47C>G) in exon 1 (GenBank accession number NM_000383), resulting in a substitution of threonine with arginine at codon 16 (p.T16R) (Fig. 1A). In addition, his parents were identified as heterozygous carriers of the same mutation (Fig. 1B).

The patient's symptoms gradually resolved after initiation of replacement therapy of glucocorticoids, corticosteroid, and levothyroxine, as well as calcium and calcitriol supplementation. He continues to do well 4 years after his hospitalization. During his last follow-up, he had serum thyroid-stimulating hormone level of 3.07 μIU/mL, free triiodothyronine level of 1.92 pg/mL, and free thyroxine level of 13.95 pg/mL. His serum cortisol and ACTH (8 a.m.) levels were 28.53 μg/dL and 69.48 pg/mL, respectively. Patient has provided informed consent for publication of the case.

## 3 Discussion

The present case showed a homozygous missense mutation p.T16R of the *AIRE* gene in a Chinese boy clinically diagnosed as APS-1, which contributes to the elucidation of the genetic background of APS-1. A search in the Human Gene Mutation Database and previous literatures indicated that this is a novel mutation. Considering that the patient's parents were asymptomatic, we can define this novel mutation as recessive. Several studies documented APS-1 caused by missense mutation p.T16M at the same amino acid position,^[7,8]^ indicating that amino acid changes at codon 16 may result conformational changes of the four-helix bundle structure in the homogenously staining region or caspase recruitment domain (HSR/CARD) of exon 1. In agreement, mutations in this domain may yield a functional defective protein that affects process of homo-/heterodimeriazation required for transcriptional transactivation activity of AIRE.^[9]^

Besides the classic triad of APS-1, the patient also had atrophic spleen as evidenced by abdominal CT scan. Based on the function of AIRE in regulating the acquisition of immune tolerance, we assumed that the etiology of spleen atrophy was an autoimmune-mediated destruction progressively leading to aspleina in a manner similar to other endocrinopathies characterizing APS-1.^[10,11]^ However, our assumption of asplenism was not supported by peripheral blood smear revealing only poikilocytosis, but none of other indicators of splenic dysfunction such as Howell-Jolly bodies, thrombocytosis, anysocites, target cells, or burr cells. Since the gradual destruction of the spleen in hypo/asplenia patients, a regular follow-up of a liver-spleen scan, and peripheral blood smear should be recommended for our patient for potential a/hyposplenism.^[10]^

Databases such as MEDLINE, Web of Science, the Cochrane Library, EMASE, CNKI, Wanfang Med Online, CiNii Articles, and airiti library were searched to explore other APS-1 patients in East Asia. A total of 29 cases of APS-1 were found up to February 2018.^[6,12–34]^ These cases as well as the one presented are reviewed in Table 1. Among 29 patients with APS-1, 8 (28%) patients had consanguineous parents. The female/male ratio was 1.5 (17 females and 11 males, 1 unknown). The median age at diagnosis was 15 years (range 8–44 years). The classic triad was found in 10 patients and a dyad was found in 17 patients (7 CMC and HP; 7 HP and AD; and 3 CMC and AD). A range of 0 to 5 minor features was presented for each patient and a high heterogeneity in clinical phenotypes was noted.

**Table 1:**
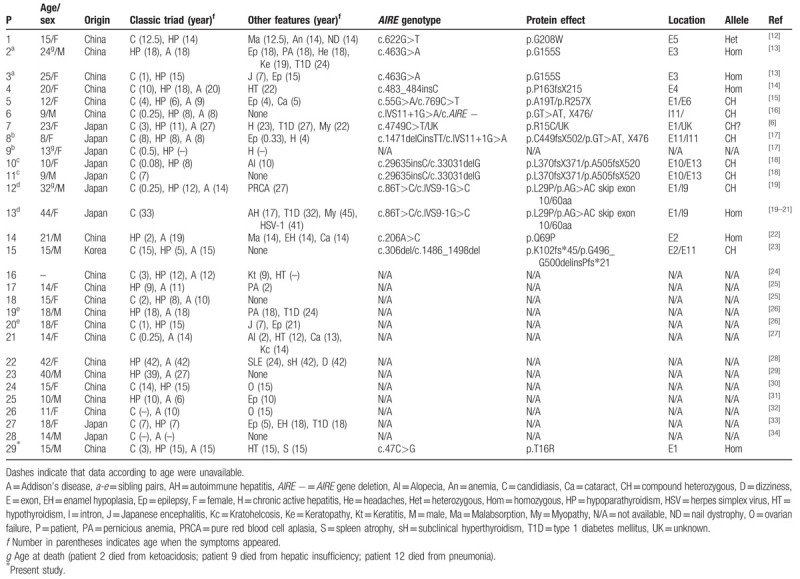
Characteristics of reviewed reports of APS-1 in East Asians.

As reported by other studies of APS-1, a couple of Japanese siblings with the same *AIRE* gene mutation displayed different phenotypes. The sister (patient 13) had three susceptible HLA loci (A 24, DRB1∗0802, and DQB1∗302) and developed T1D at 32-year-old, whereas her younger brother (patient 12) had protective allele DQB1∗0602 for T1D was absence of T1D. Likewise, patients with same clinical features may be caused by different *AIRE* mutations. One patient with pure red blood cell aplasia had a compound heterozygous p.L29P and c.IVS9-1G>C mutation in the *AIRE* gene. While the disease was also seen in one Russian and one Serbian patient both with homozygous R257X mutation.^[35,36]^ In our study, there were some special cases of APS-1. Systemic lupus erythematosus (SLE) coexisting with APS-1 was reported in one Chinese female with APS-1 (patient 22). Her clinical manifestations were SLE, hypoparathyroidism, AD, and subclinical hyperthyroidism. To the best of our knowledge, this was the first case report of APS-1 patient co-presenting with an SLE diagnosis. A study demonstrated the presence of testis specific, 10 autoantibodies in APS-1 and SLE patients, but whether SLE is a component of APS-1 is yet to be identified.^[37]^ Furthermore, chromosome analysis of a 15-year-old girl (patient 18) revealed a 46, XX/46, XY (3:1) karyotype. Her menarche started at 13-year-old and has female genitalia. The development of breasts and pubic hair was in Tanner stage II. Pelvic ultrasound revealed normal uterus and ovaries. It is unlikely that this patient is a true hermaphroditism. Other minor components such as pure red blood cell aplasia, subclinical hyperthyroidism, and muscular dystrophy have been reported by other studies.

We also compared the prevalence of clinical components with the results of previous studies in Finland,^[5]^ Iranian Jews,^[3]^ Italy,^[38]^ Norway,^[39]^ Russia,^[40]^ and North-western France^[41]^ (Table 2). Generally, CMC is the most frequent clinical manifestations among the classical triads and appear earliest in life as early as the first month after birth and often occurring before age 5. However, CMC was the second most frequent manifestations observed in 22 (76%) East Asian patients. The median age of onset for CMC was 3 years (range 0.08–33 years), which was in consistence with other ethnic groups. Hypoparathyroidism is the most common endocrine component in APS-1 patients and usually appears after CMC with the incidence peaks at age 2–11 years. In our study, hypoparathyroidism was the most prevalent feature seen in 24 (83%) patients with the median age of 15 years (range 6–42 years). AD is generally the second most common endocrine disorder in patients with APS-1 with the peak incidence around 12 years of age. Similarly, AD was found in 20 (69%) patients with the median age at onset of 14 years (range 6–42 years). The most three frequent minor manifestations were autoimmune thyroiditis (17%) with the median age of onset at 18.5 years (range 12–42 years), TID (17%) with the median age of onset at 24 years (range 18–32 years), and hepatitis (14%) with median age of onset at 17 years (range 4–23 years). We found that the frequency of autoimmune thyroiditis (17%) was higher in East Asia than in other countries (4–10%) and similar to Russia (20%). Likewise, T1D (17%) was more prevalent compared to other countries (2–12%). The frequency of ovarian failure (7%) was same in East Asia and Russia, but lower than in other countries (38–71%). Moreover, alopecia (7%) was less likely to occur in East Asians, but was more common in other countries (13–53%).

**Table 2:**
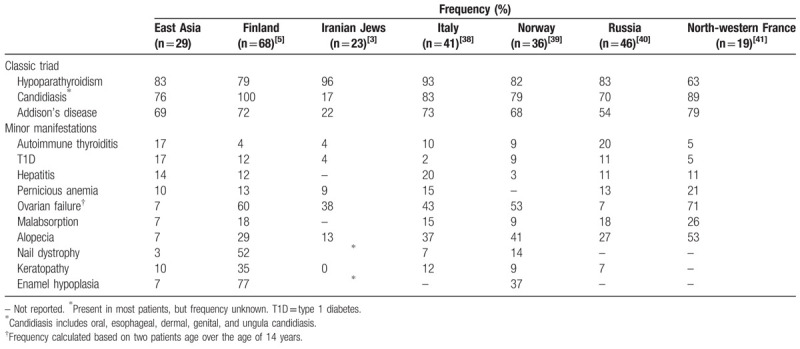
Prevalence of the main clinical components of APS-1 in different countries.

**Figure 2:**
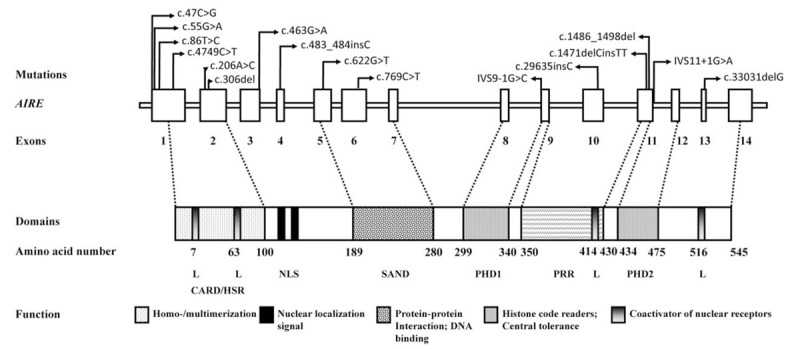
Schematic representation of the *AIRE* gene and protein and the position of APS-1 mutations in East Asian patients. HSR/CARD, homogenously staining region or caspase recruitment domain/homodimerization domain; NLS =  nuclear localization signal, SAND = *S*p100, *A*IRE, *N*ucP41/75 and *D*EAF-1; PHD = plant homeodomain zinc finger, PRR = proline-rich region, L = the LXXLL nuclear receptor interaction motif.

In total, 16 different mutations were identified in the *AIRE* gene in 15 patients came from 12 different families (Table 1). Compound heterozygous (46%) and homozygous (40%) mutations were more common. One heterozygote p.G208W (patient 1) that may act in a dominant fashion. *AIRE* mutation hot spots seem to be different in specific geographic regions and ethnic groups. However, *AIRE* mutations in East Asian patients appeared to be greatly diverse and no predominant mutation was found (Fig. 2). Although majority of the studies reported no genotype-phenotype correlation in APS-1, a few observations suggested that certain *AIRE* mutations or polymorphisms were associated with common autoimmune diseases. For example, c.11107G>A may be involved in the pathogenesis of systemic sclerosis associated with autoimmune thyroiditis.^[42]^ Yet, the association was not observed in our patients 7 with the same single nucleotide polymorphisms (data not shown), suggesting environmental factors as well as other genetic elements besides the *AIRE* gene modify the variety of phenotypes in APS-1 patients.

## 4 Conclusions

We herein report a novel missense mutation in the *AIRE* gene in a Chinese boy with APS-1 accompanied by atrophic spleen and autoimmune thyroiditis. APS-1 is rare in East Asian population and the variable phenotypes make the initial diagnosis especially difficult. We summarized 29 APS-1 cases in East Asia and found that the characteristics of classic triad such as prevalence and age of onset resemble other European countries, but East Asian patients with APS-1 were more likely to complicate with autoimmune thyroiditis and T1D, while less likely to have ovarian failure, malabsorption, and alopecia. No prevalent *AIRE* mutations were noted. Although a large-scale statistical analysis and research is necessary before a final conclusion can be made, our study demonstrated characteristics of APS-1 in East Asian populations for clinical practice and future studies.

## Author contributions

**Conceptualization:** Zi Yan, Zhuo Li.

**Data curation:** Zi Yan, Xiaona Xie, Ying Gao.

**Funding acquisition:** Xiaokun Gang, Guixia Wang.

**Investigation:** Xiaona Xie, Ying Gao.

**Methodology:** Xiaokun Gang, Zhuo Li.

**Supervision:** Guixia Wang.

**Writing – original draft:** Zi Yan.

**Writing – review & editing:** Xiaokun Gang, Guixia Wang.

Guixia Wang orcid: 0000-0001-8107-616X.
